# Local mechanical behavior and stress-driven evolution of lithium dendrites revealed by *operando* electrochemical liquid-cell TEM

**DOI:** 10.1039/d6mh01280a

**Published:** 2026-09-02

**Authors:** Walid Dachraoui, Ruben-Simon Kühnel, Corsin Battaglia, Rolf Erni

**Affiliations:** a Electron Microscopy Center, Empa––Swiss Federal Laboratories for Materials Science and Technology Überlandstrasse 129 8600 Dübendorf Switzerland walid.dachaoui@empa.ch rolf.erni@empa.ch; b Materials for Energy Conversion, Empa––Swiss Federal Laboratories for Materials Science and Technology Überlandstrasse 129 8600 Dübendorf Switzerland; c Department of Information Technology and Electrical Engineering––ETH Zürich Gloriastrasse 35 8092 Zürich Switzerland; d Institute of Materials, School of Engineering – EPFL Station 15 1015 Lausanne Switzerland; e Department of Materials – ETH Zürich Wolfgang-Pauli-Strasse 10 8049 Zürich Switzerland

## Abstract

The formation of Li dendrites in lithium metal batteries (LMBs) poses major safety risks and degrades performance through continuous active lithium consumption. Understanding their growth and evolution is therefore critical for improving battery efficiency and long-term stability. Here, we investigate Li dendrite dynamics using *operando* electrochemical liquid-cell transmission electron microscopy (ec-LC-TEM), enabling direct nanoscale observation during electrochemical cycling. Our results demonstrate that dendrite growth is not governed solely by diffusion-controlled root- or tip-growth mechanisms but evolves toward a stress-assisted local Li deposition regime driven by mechanical interactions between neighboring Li dendrite structures. Li dendrites in the form of whiskers formed *via* root growth undergo stress-induced deviations, leading to contact, coalescence, and loop formation. These loop-like structures possess two active ends that serve as opposing Li deposition sites, generating growth in opposite directions and producing localized compressive stress. This stress promotes SEI cracking, the formation of new electrochemically active sites, and defect generation, enabling internal Li transport and mass redistribution. Together, these coupled electrochemical–mechanical processes accelerate dendrite growth, drive folding, and govern the transition toward multi-site structural evolution. These findings provide a revised mechanistic framework for Li dendrite growth and offer new opportunities for suppressing dendrites in LMBs.

## Abstract 2

Mechanical constraint transforms Li dendrite growth: *operando* liquid-cell TEM reveals how SEI disruption and localized Li deposition redirect root growth toward stress-assisted loop formation, folding, and multidimensional structural evolution.

## Graphical abstract

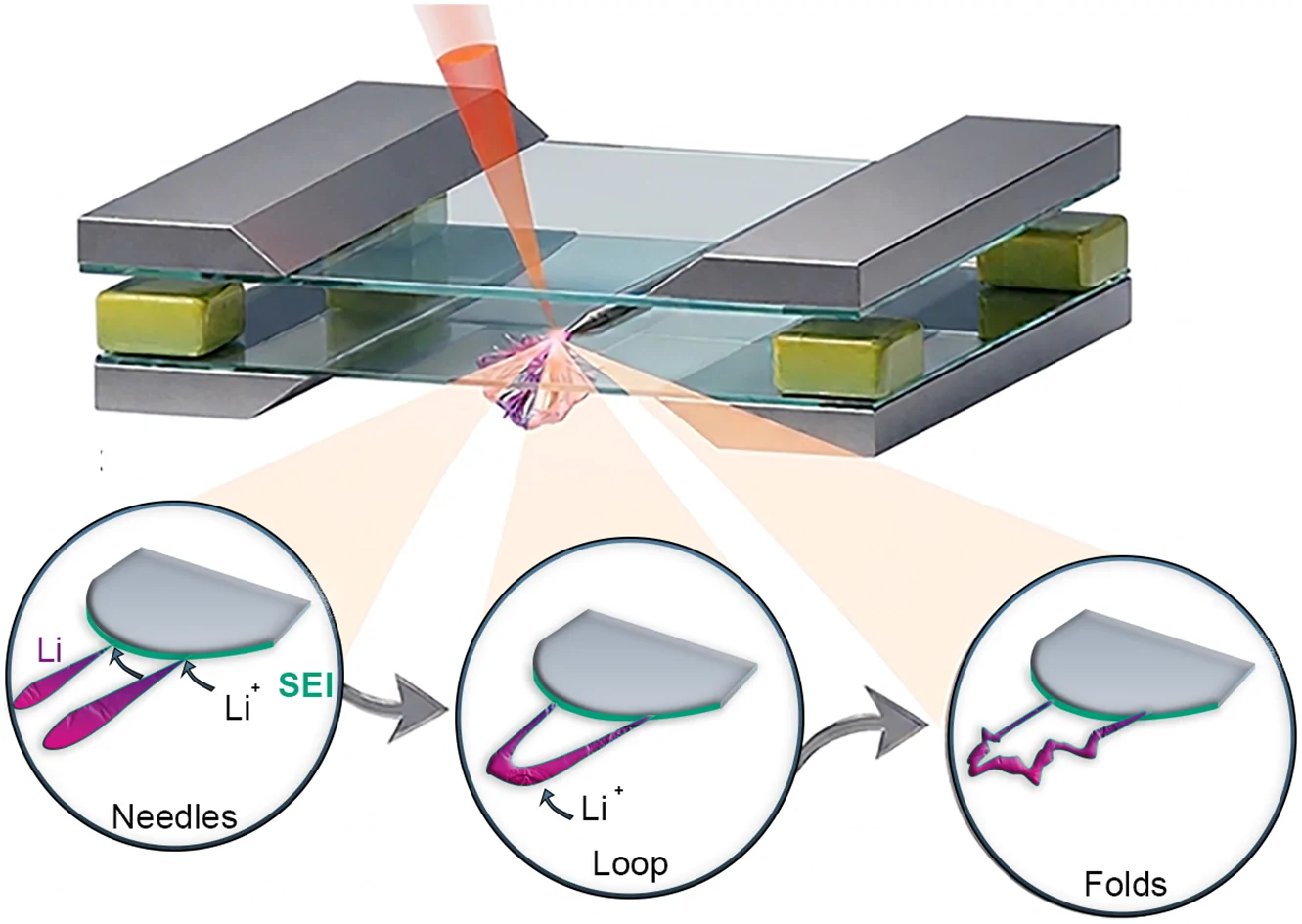

**New concepts**

Lithium dendrite growth remains one of the major challenges limiting the practical implementation of Li metal batteries (LMBs) and is generally described by slow diffusion-controlled root- or tip-growth mechanisms governed by electrochemical and solid electrolyte interphase (SEI) properties. Here, we reveal a previously unrecognized electrochemo-mechanical growth mechanism in which dendrite evolution transitions from a diffusion-limited regime to a significantly faster stress-assisted local deposition regime. Using *operando* electrochemical liquid-cell transmission electron microscopy, we demonstrate that mechanical interactions between neighboring Li whiskers lead to loop-like structures with multiple active growth ends. The resulting multidirectional growth generates localized compressive stress, which promotes SEI cracking and creates new electrochemically active sites for Li insertion. This stress-driven activation accelerates local Li deposition, giving rise to plastic deformation, and multi-site growth that cannot be explained by conventional diffusion-controlled dendrite models. By identifying mechanical stress as an active driving force for Li deposition, rather than merely a consequence of dendrite growth, this work establishes a revised framework for Li dendrite evolution and introduces new opportunities to engineer mechanically and electrochemically robust interfaces for suppressing dendrite formation and enabling safer, longer-lasting LMBs.

## 1. Introduction

Lithium metal anodes are widely regarded as a key technology for next-generation lithium metal batteries (LMBs) due to their exceptionally high theoretical capacity (3860 mA h g^−1^) and low electrochemical potential (−3.04 V *vs.* standard hydrogen electrode (SHE)).^1,2^ These attributes enable LMBs with energy densities far exceeding those of conventional lithium-ion batteries (LIBs).^3^ However, their practical implementation is fundamentally hindered by the formation of Li dendrites during repeated charge–discharge cycling.^4^ These filamentary structures grow uncontrollably from the Li metal surface, leading to internal short circuits, continuous electrolyte consumption, and rapid degradation of cell performance, ultimately posing severe safety risks.^5–7^ Over the past decades, considerable efforts have been devoted to mitigating dendrite formation through electrolyte engineering, artificial interphase design, current regulation, and electrode structuring.^8–10^ Although these strategies have led to notable improvements in cycling stability, they largely rely on empirical optimization rather than a predictive understanding of dendrite behavior. An unresolved challenge is to elucidate the fundamental mechanisms governing Li dendrite nucleation, propagation, and morphological evolution, particularly at the nanoscale, where electrochemical, mechanical, and interfacial processes are strongly coupled and dynamically evolving.^10–12^

Addressing these challenges requires a fundamental understanding of the dynamic processes occurring at the anode–electrolyte interface. Achieving this objective necessitates real-time observation of interfacial evolution during battery operation through *in situ* and *operando* characterization techniques. Among the available approaches, *operando* transmission electron microscopy (TEM) employing an electrochemical liquid cell, has emerged as a powerful platform due to its unique ability to provide high spatial and temporal resolution during electrochemical cycling.^13–16^

*Operando* electrochemical liquid-cell TEM (ec-LC-TEM) has been extensively utilized to investigate dynamic mechanisms in LMBs, including SEI formation and its evolution, cathode material transformations, and the growth and dissolution of Li dendrites.^17–22^ Recent studies have particularly leveraged this approach to elucidate Li dendrite growth mechanisms with high spatio-temporal resolution. For example, Nishikawa *et al.* reported a linear relationship between length of Li deposits and the square root of time, attributing this behavior to diffusion-limited ion transport within the electrolyte.^23^ More recently, Kushima *et al.* employed ec-LC-TEM to investigate Li metal deposition on Au electrodes *via* a root-growth mode, proposing a mechanism governed by competition between Li deposition and SEI formation, in which Li atoms deposit beneath the SEI and drive whisker growth from the root.^24^ Building on these insights, our recent *operando* TEM investigations demonstrated that Li growth follows a more complex multi-step process, initially governed by SEI-controlled root growth and subsequently transitioning to a tip-driven mechanism following SEI fracture, which enables branch formation and dendritic structure development.^25^

Despite significant progress, current descriptions of Li dendrite growth remain incomplete, as dendrite evolution is often simplified to isolated whisker growth while neglecting the surrounding environment and dynamic interactions between neighboring Li structures. This oversimplification obscures the critical role of mechanically coupled electrochemical processes in governing dendrite morphology. In particular, how Li dendrites interact upon contact, reorganize under mechanical stress, and evolve into deformed or kinked architectures remains poorly understood. Elucidating these mechanisms is essential for the rational design of electrodes, electrolytes, artificial SEI layers, and separators to more effectively mitigate the detrimental impact of Li dendrites. To address these questions, we employ *operando* ec-LC-TEM to directly probe the nanoscale evolution of Li dendrites during electrochemical cycling. By resolving dendrite dynamics in real time, we uncover how local interfacial heterogeneity, SEI evolution, and mechanical interactions collectively govern structural reconfiguration beyond conventional isolated whisker models. Our study identifies the mechanistic pathways linking early-stage whisker growth to subsequent structural deviation, interconnection, and deformation, with particular emphasis on how neighboring Li structures interact, reorganize, and evolve under stress. Through this approach, we establish a broader framework that connects electrochemical deposition, mechanical response, and structural complexity in Li dendrite evolution, providing new insight into the origins of dendritic architectures and their implications for battery degradation.

## 2. Materials and methods

### 2.1 *In situ* electrochemical liquid cell TEM setup

**Fig. 1:**
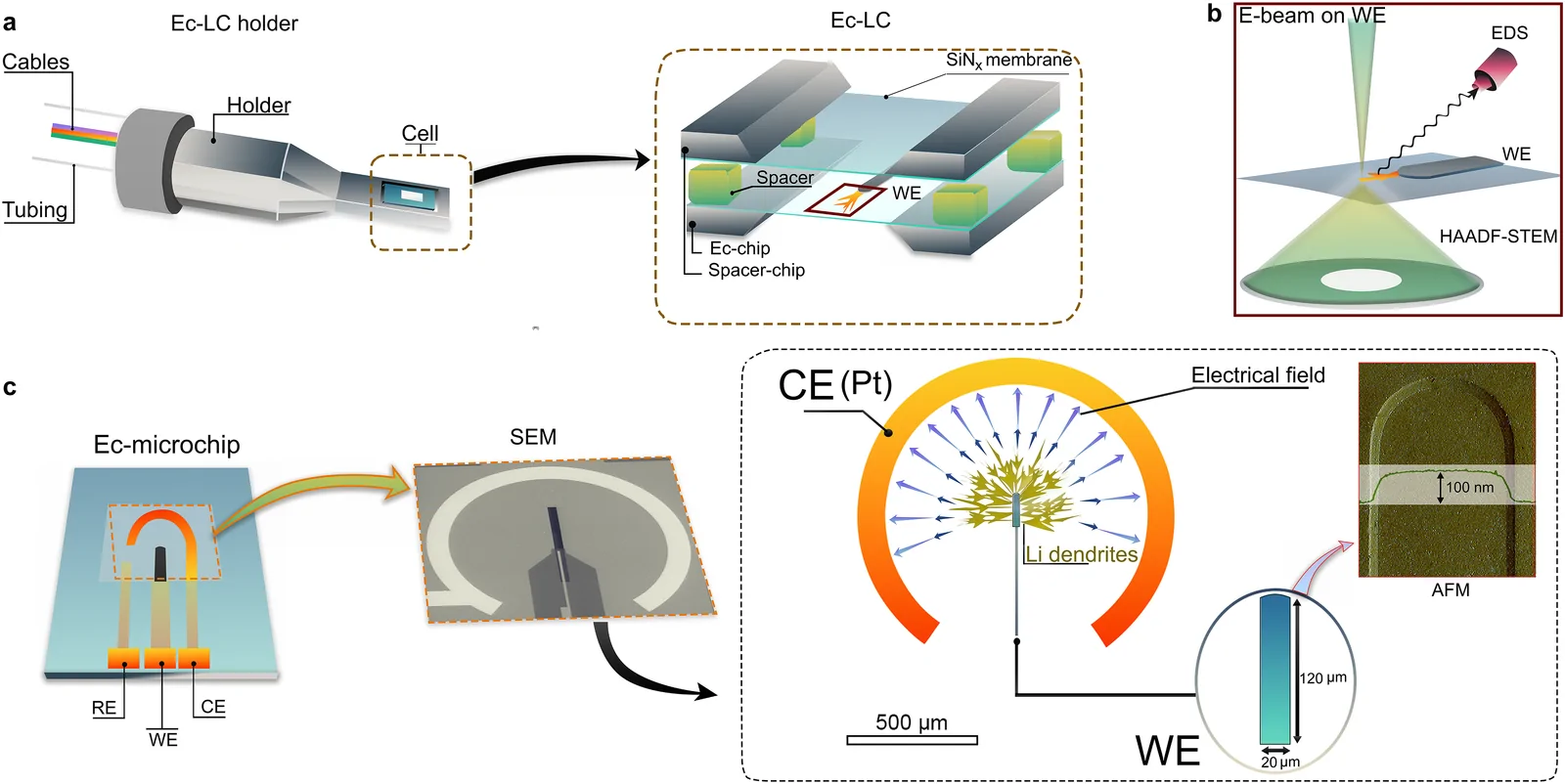
*In situ* electrochemical liquid-cell TEM setup for lithium dendrite growth experiments. (a) Schematic of the tip of the holder and assembled liquid cell, showing stacked microchips with SiN_*x*_ membranes, spacer, and WE. (b) Illustration of e-beam interaction enabling analysis on the WE. (c) Microchip electrode layout (RE, WE, CE) with corresponding SEM image focusing on WE and CE. The semicircular CE generates a quasi-radial electric field, promoting lithium deposition from the WE toward the CE; inset shows representative dendrite formation.

Fig. 1 provides an overview of the ec-LC-TEM holder, liquid-cell architecture, and electrode configuration used for Li dendrite growth. Experiments were performed using a Poseidon 510 holder (Protochips Inc.)^26^ integrated into a probe-corrected Titan Themis TEM operated at 300 kV. The liquid cell is formed by stacking two silicon-based microchips sealed with an O-ring system (Fig. S1). Both chips contain ∼50 nm thick electron-transparent silicon nitride (SiN_*x*_) membranes that confine the electrolyte within a few hundred nanometer wide gap compatible with *in situ* TEM imaging.^26–28^ The liquid thickness is controlled by spacers located on the bottom spacer-chip together with membrane thickness affecting membrane bulging under vacuum conditions.

The top ec-chip incorporates three patterned electrodes: Pt reference (RE) and counter (CE) electrodes, and a glassy carbon (GC) working electrode (WE) positioned at the center of the SiN_*x*_ membrane, defining the primary imaging region (Fig. 1b). GC was selected because of its electron transparency, chemical stability, and minimal volume variation during electrochemical cycling, enabling stable observation of the electrode/electrolyte interface, SEI evolution, and Li dendrite growth.^29^ As illustrated in Fig. 1c, the CE is designed as a semicircular ring surrounding the WE to promote a homogeneous quasi-radial electric field distribution and minimize local polarization effects.^30^ This geometry enables Li deposition and subsequent growth from the glassy carbon WE into the surrounding electrolyte-filled region, providing a controlled platform to study the directional growth and morphological evolution of Li dendrites under electrochemical conditions.

The complete configuration used to investigate Li dendrite formation is summarized in Fig. S1. After assembly of the electrochemical liquid cell, the holder was inserted into the transmission electron microscope following prior validation of the liquid-cell performance (see SI and Fig. S1 for detailed assembly). The on-chip electrodes were connected to an external potentiostat *via* dedicated electrical feedthroughs, enabling controlled electrochemical measurements during imaging. The electrolyte was delivered through polyether ether ketone (PEEK) tubing at a constant flow rate of ∼1 µL min^−1^ using a syringe pump, ensuring a stable liquid environment throughout the experiment. A standard electrolyte (1 M LiPF_6_ in EC : EMC (3 : 7 vol)) was employed. Prior to *operando* measurements, the system was systematically tested to confirm reliable electrical contact between the electrodes and proper communication with the potentiostat, ensuring stable and reproducible electrochemical control during *operando* scanning (S) TEM observations.^31^

## 3. Results and discussion

### 3.1. Li dendrites growth on the microchip

**Fig. 2:**
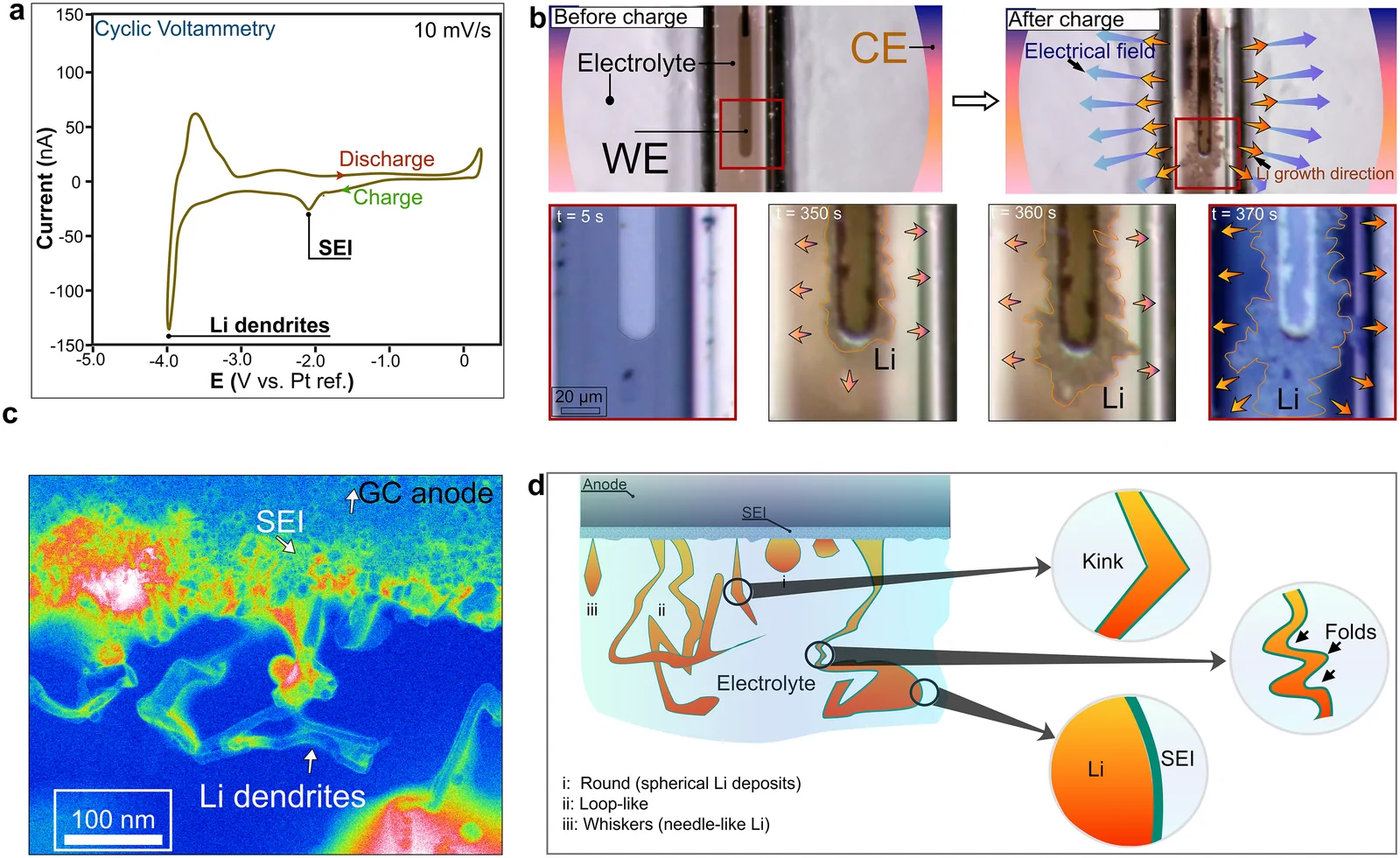
Multiscale observation of Li plating and dendrite growth on the GC electrode. (a) Cyclic voltammetry showing SEI formation and Li deposition/stripping peaks. (b) Optical microscopy images of the GC electrode before and after charging, with time-resolved snapshots illustrating Li growth from the electrode edge and its alignment with the local electric field. (c) *In situ* STEM image revealing nanoscale morphology of Li dendrites and the SEI at the GC–electrolyte interface. (d) Schematic illustration summarizing Li growth deposits, including spherical structures, branched dendrites, and whisker-like structures, as well as stress-driven deformation and folding within the dendrites.

Cyclic voltammetry (CV) was employed to probe Li plating on the GC electrode under a constant electrolyte flow rate of 1 µL min^−1^ and a scan rate of 10 mV s^−1^. As shown in Fig. 2a, the cathodic sweep exhibits two distinct reduction features. A first, minor peak at approximately −2.0 V *vs.* Pt is attributed to electrolyte decomposition and the formation of the SEI layer.^32^ This is followed by a pronounced peak near −4.0 V *vs.* Pt, corresponding to Li metal deposition.^25^ During the reverse scan, a stripping peak appears around −3.8 V *vs.* Pt, associated with Li dissolution. The following discussion focuses on Li nucleation, growth, and the associated mechanical behavior of Li during deposition.

Prior to *in situ* STEM imaging of the GC–electrolyte interface at the nanoscale, optical microscopy (OM) was employed to assess the overall morphology and growth direction of Li deposits. As shown in Fig. 2b (top panels), the GC electrode initially exhibits a well-defined, sharp edge, which evolves significantly after the charging process. Li deposits preferentially form at the electrode edge due to geometrically induced field enhancement. The most pronounced growth is observed at the electrode tip, consistent with the locally enhanced current density in this region.^33^ Time-resolved OM observations reveal that Li growth initiates at the GC edge as a thin, shell-like layer that progressively thickens and extends into the electrolyte. With continued deposition, distinct protrusions emerge and develop into coral-like branches aligned with the local electric field (blue arrows in Fig. 2b). These observations confirm that Li dendrite growth is strongly governed by the electric field distribution, promoting directional extension from the electrode edge into the electrolyte. Notably, the close spatial proximity of neighboring dendrites suggests a high likelihood of interaction, including collision and stress-induced contact, which may play a critical role in their subsequent morphological evolution.

To further resolve the growth processes at the nanoscale, *operando* STEM imaging of the GC–electrolyte interface was performed. As shown in annular dark-field scanning transmission electron microscopy (ADF-STEM) images (temperature-contrast rendering) in Fig. 2c, Li deposition leads to the formation of complex dendritic structures emerging from the electrode surface and extending into the electrolyte. The interface exhibits a heterogeneous morphology, where a relatively continuous SEI layer covers the GC surface, while Li deposits nucleate beneath and grow through this interphase, consistent with our previous observations.^25^ The resulting dendrites display irregular, branched, and filamentary geometries with pronounced variations in thickness and curvature, indicative of a highly dynamic growth process.

At higher magnification (Fig. 2c), multiple Li morphologies are observed, including compact deposits, mossy-like structures, loop-like features, and elongated whiskers. These observations indicate that Li growth proceeds through localized nucleation events followed by anisotropic extension into the electrolyte. The coexistence of these distinct morphologies reflects spatial variations in local electrochemical conditions, interfacial stability, and ionic transport. More importantly, detailed examination of elongated whiskers formed during charging reveals pronounced deformations, including kinks and folds along their length. Such features cannot be explained solely by electrochemical factors and instead indicate a significant mechanical contribution to the growth process. In particular, the observed folded morphologies suggest the development of internal stresses during Li deposition, leading to plastic deformation of the dendritic structures. Understanding the origin and evolution of these mechanically driven instabilities is therefore essential for a comprehensive description of Li dendrite growth.

A schematic representation summarizing the range of observed Li morphologies is presented in Fig. 2d, constructed based on the ADF-STEM observations in Fig. 2c and complementary datasets (Fig. S2). The schematic illustrates the coexistence of spherical deposits, branched dendrites, and whisker-like structures, as well as the emergence of stress-induced features such as folding, providing a unified framework for interpreting Li growth behavior under the investigated conditions.

### 3.2. Growth and stress-driven mechanical behavior of Li dendrites

**Fig. 3:**
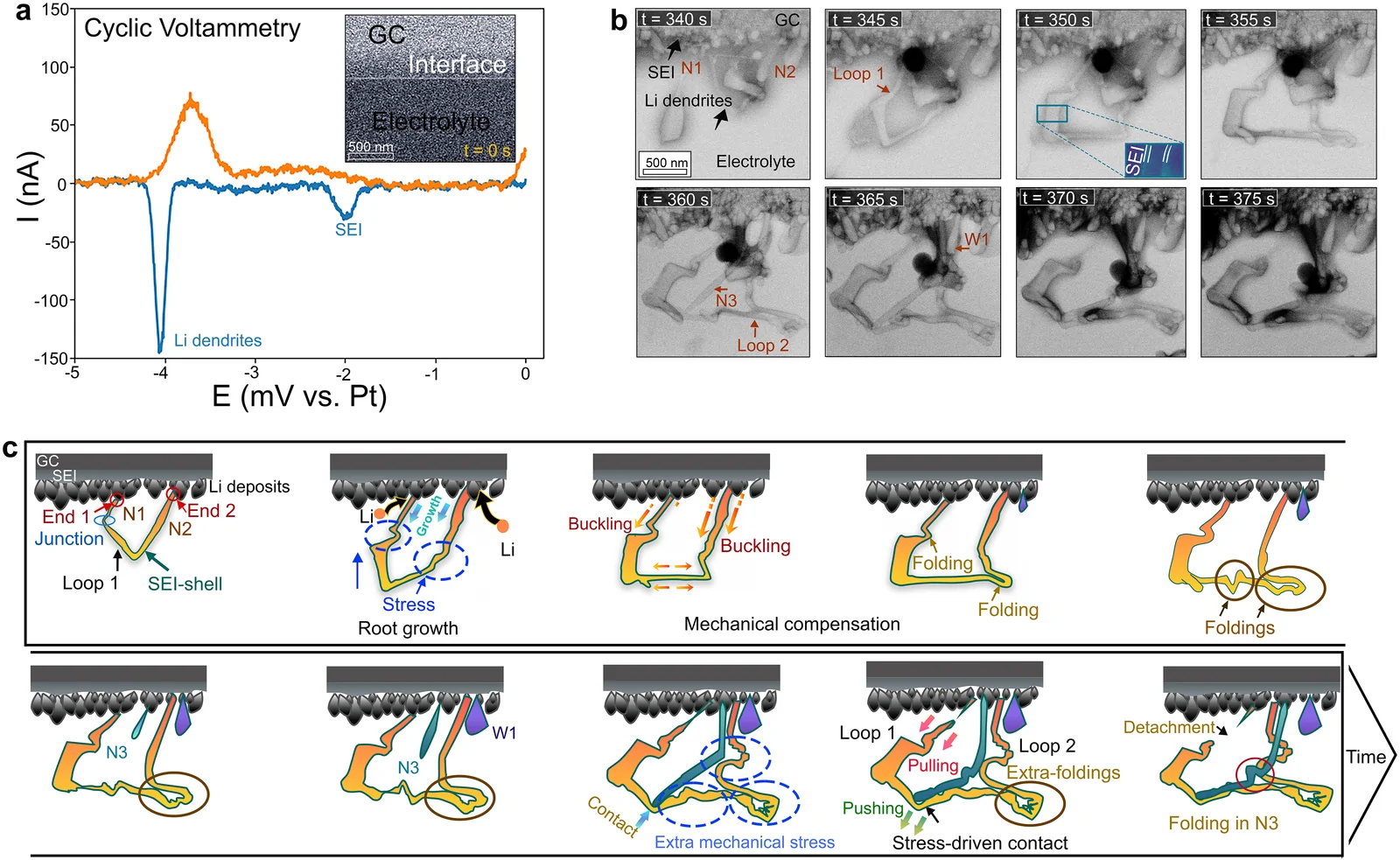
Local nucleation, growth, and stress-driven mechanical evolution of Li dendrites at the GC–electrolyte interface during *in situ* electrochemical cycling. (a) Cyclic voltammogram recorded during real-time ADF-STEM imaging, with the inset indicating the initial imaging region prior to cycling. (b) Time-lapse ADF-STEM image sequence (inverted contrast) showing the progressive formation and deformation of lithium dendrites. (c) Simplified mechanistic schematic summarizing the proposed electrochemo-mechanical pathway governing lithium whisker evolution under continued root-driven growth and local mechanical constraints.

To elucidate the mechanisms governing the diversity of Li morphologies and their associated deformations, we monitored the GC working electrode–electrolyte interface at high spatio-temporal resolution during voltametric cycling. Fig. 3a presents a representative cyclic voltammogram acquired during *in situ* electrochemical cycling under real-time ADF-STEM imaging. The corresponding imaging region is indicated in the top-right inset (*t* = 0 s), where the GC edge is initially smooth and well-defined prior to electrochemical activation. Fig. 3b shows a time-lapse series of ADF-STEM images extracted from movie S1 (displayed with inverted contrast for clarity) from the same region during Li deposition (associated with the large cathodic peak around −4 V *vs.* Pt).

At *t* ≈ 340 s, corresponding to the onset of Li plating, the first Li deposits become visible. The ADF-STEM images are governed by mass–thickness contrast, where intensity increases with material density and thickness. Consequently, Li appears with lower (darker) contrast relative to the surrounding phases; contrast inversion is therefore applied to enhance dendrite visibility and facilitate morphological tracking.^34^ The ADF-STEM observations further reveal that Li structures are encapsulated by a thin outer shell with relatively higher contrast (clearly visible in the zoomed region at *t* ≈ 350 s in Fig. 3b), consistent with an SEI surrounding the growing Li (hereafter referred to as the SEI-shell). This SEI-shell plays a central mechanical role in the evolution of the structures.^25^

At *t* ≈ 340 s, a Li structure exhibiting multiple kinks and anchored at two points within the SEI becomes evident. This structure grows more rapidly than the surrounding elongated needles and shorter whiskers and is hereafter referred to as a loop (Loop1). For clarity, the long, high-aspect-ratio Li structures are denoted as needles (*N*), while the shorter, rounded deposits are referred to as whiskers (*W*).

From approximately *t* ≈ 350 s, Loop1 develops pronounced kinks and folding, which intensify with continued growth. Around *t* ≈ 360 s, a new needle (N3) emerges and grows rapidly until it contacts Loop1, inducing deformation and eventual attachment. Following this interaction, N3 also undergoes folding. In contrast, the shorter whiskers exhibit significantly less dynamic behavior; one representative whisker (W1) is selected to illustrate their comparatively slow growth mechanism.

To better rationalize the processes occurring within this localized region, a schematic illustration is constructed to describe the evolution of Loop1 formed initially *via* the interaction of two initial needles (N1 and N2), as well as the subsequent evolution of N3 and W1. Loop1 initially forms as a closed structure anchored at two ends (End 1 and End 2), corresponding to the roots of N1 and N2. This configuration establishes two active Li deposition sites, enabling growth toward the electrolyte from both ends. The simultaneous deposition generates compressive stress within the loop, which is accommodated through mechanical responses including thickening, buckling, and ultimately folding.

As Li deposition continues at both ends, facilitated by incomplete SEI densification, the loop undergoes progressive folding to relieve the accumulated stress. Concurrently, a slower growth regime develops in the surrounding region, where mossy-like structures evolve gradually. In parallel, N3 grows rapidly and, through directional deviation and kink formation, eventually contacts Loop1, exerting additional mechanical stress and pushing the loop toward the electrolyte. This interaction forms a junction between the two structures, leading to the emergence of a secondary loop (Loop2). The resulting mechanical interaction induces further deformation of Loop1, including partial detachment at one end due to tensile stress along N1. Subsequently, Loop2 continues to grow from two active ends (End 2 and the root of N2), undergoing additional folding in response to the sustained compressive stress generated by bidirectional growth. Meanwhile, the mossy-like deposits (W1) evolve more slowly and independently in parallel with the dominant loop and needle structures. Although the observed behavior is primarily attributed to the interplay between electrochemical deposition, SEI evolution, and mechanically induced stress, the geometric confinement imposed by the SiN_*x*_ membranes may provide an additional contribution by restricting out-of-plane deformation. However, because the electrode configuration promotes Li growth predominantly within the plane parallel to the SiN_*x*_ membranes, this confinement effect is expected to play only a secondary role and is unlikely to be the dominant source of the observed stress accumulation. Overall, this sequence reveals a highly coupled growth mechanism in which electrochemical deposition, SEI evolution, and mechanical stress collectively govern lithium dendrite morphology.

These observations raise fundamental questions regarding the driving forces underlying stress-induced folding and structural reconfiguration, highlighting the need to consider the interplay between electrochemical processes and nanoscale plastic deformation in lithium metal systems.

### 3.3. What drives Li dendrites folding and deformation?

**Fig. 4:**
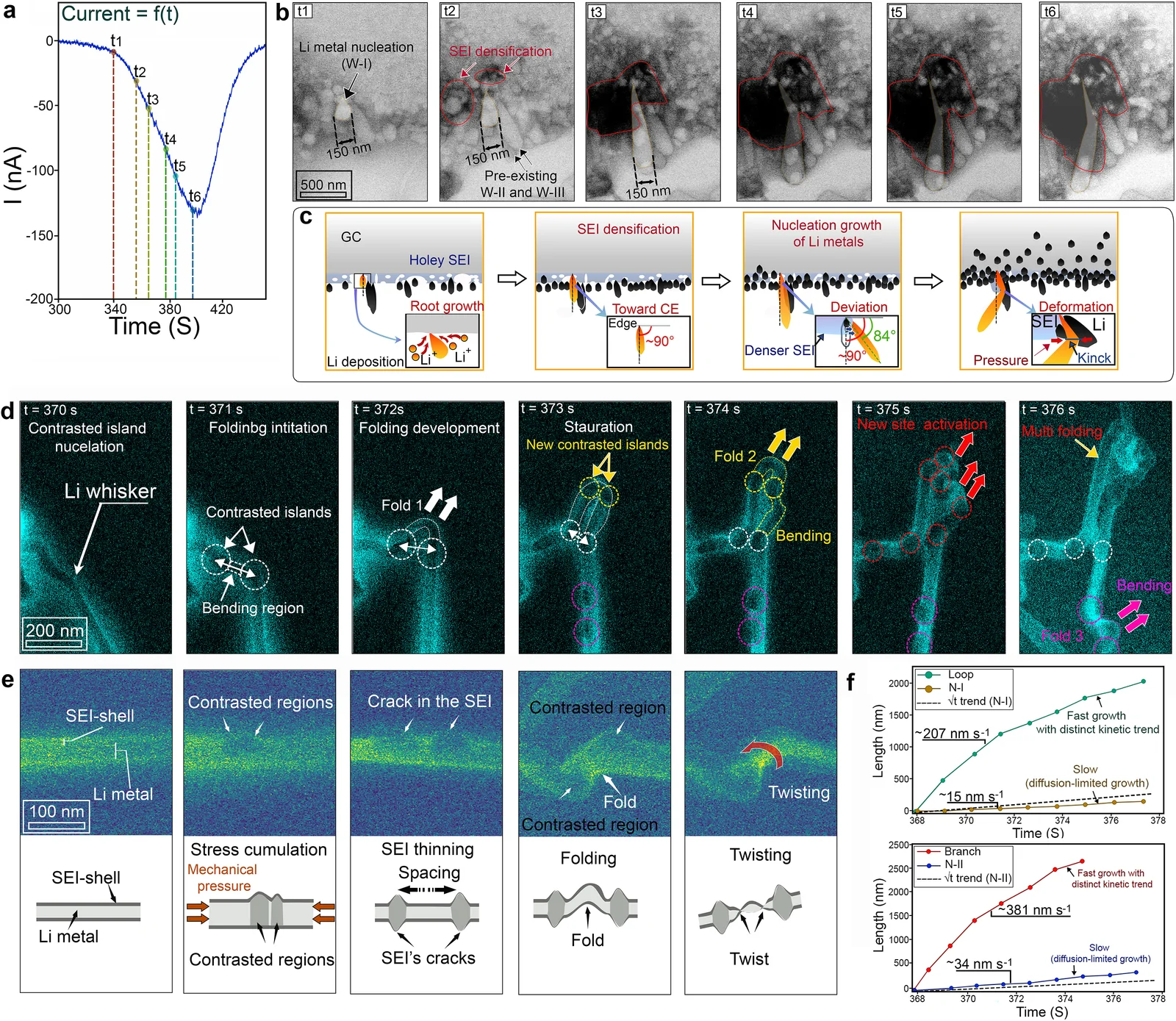
Time-resolved ADF-STEM investigation of lithium dendrite evolution. (a) Current–time profile during Li electrodeposition with selected time points. (b–e) Corresponding time-lapse images and mechanistic interpretation showing nucleation, SEI densification, whisker deviation, kink formation, folding, and stress localization. (f) Quantitative growth kinetics comparing Loop *vs.* needle (top) and branch *vs.* needle (bottom), highlighting the transition from diffusion-limited growth to stress-assisted, multi-site growth.

To identify the fundamental driving forces underlying Li dendrite folding and deformation, it is essential to resolve the early local events that precede large-scale structural instability. Fig. 4a presents the current–time profile corresponding to the Li electrodeposition peak, with selected time points (*t*_1_–*t*_6_) highlighted. These time points are directly correlated with the time-resolved ADF-STEM images shown in Fig. 4b (extracted from Movie S2), enabling a direct link between electrochemical response and morphological evolution at the nanoscale.

As shown in Fig. 4b (*t*_1_*–t*_6_), prior to the onset of large-scale folding, the first critical event is the nucleation of small, spherical Li deposits. One representative particle, denoted W-I, is selected to follow its evolution during the charging process. Between *t*_1_ and *t*_2_, as the potential becomes more negative, this particle is progressively displaced away from the GC edge toward the electrolyte, accompanied by the deposition of a thinner Li layer beneath it. With continued deposition, this process leads to the formation of an elongated whisker characterized by a relatively constant tip diameter (∼150 nm) and a thinner root, consistent with a root-based growth mechanism.^25^

At this early stage, Li deposition remains relatively localized. However, a striking transformation occurs with the emergence of distinct contrasted “islands” near the whisker and along the surrounding interphase. These regions progressively intensify prior to the onset of major deformation, indicating that they correspond to localized structural or mechanical heterogeneities rather than simple lithium extension. Based on their higher contrast and interfacial localization, these features are consistent with local SEI densification or mechanically reinforced SEI domains. Such densified regions act as rigid pinning points that progressively constrain W-I, inducing a gradual deviation in its growth direction from approximately 90° relative to the GC edge to about 84°. As growth proceeds, the whisker tip interacts with pre-existing neighboring whiskers (W-II and W-III), forcing further directional deviation, as illustrated in the schematic in Fig. 4c. This interaction generates localized kinks that enable the whisker to propagate along new directions. Simultaneously, continued SEI densification and Li deposition at the root introduce additional stress sources, reinforcing directional instability and promoting further kink formation. Through successive directional changes, neighboring Li needles are brought into contact, ultimately leading to loop formation.

Although the formation of structures connected to the electrode at both ends cannot be directly resolved at the earliest stages, loops become clearly visible at later stages of deposition. Based on these observations, combined with the mechanism proposed for Loop2 in Fig. 3, we infer that loop formation arises from the interaction and merging of adjacent whiskers. Once formed, these loops possess two active ends that act as Li deposition sources, enabling bidirectional growth. This configuration introduces opposing growth directions, generating internal mechanical constraints within the structure. As a result, the loop undergoes progressive deformation and folding, the origin of which requires further elucidation.

Fig. 4d presents an ADF-STEM time-lapse sequence of a selected loop region, capturing the onset and evolution of folding during growth. At *t* = 370 s, the selected segment of the Li whisker appears relatively straight and is uniformly surrounded by an SEI-shell, exhibiting a homogeneous contrast. Between *t* = 370 s and *t* = 371 s, a sudden formation of two neighboring contrasted islands is observed, separated by a narrow region of lower contrast, which corresponds to a localized bending zone.

At *t* = 372 s, a fold begins to form at this location, and the deformation propagates along the whisker with time. By *t* = 373 s, the initial folding region reaches a saturation stage, accompanied by the emergence of new contrasted islands at the whisker tip (highlighted by yellow dashed circles). These newly formed regions act as nucleation sites for additional bending, initiating subsequent folding events between adjacent islands. This process repeats progressively, leading to the development of a multi-folded region. Notably, the folding does not remain localized but migrates along the whisker through the sequential appearance of new contrasted islands, indicating a dynamic redistribution of stress and the activation of new deformation sites along the structure. As deformation proceeds, folding is initiated at these stress concentration zones and subsequently propagates along the whisker, leading to multiple folding events and curvature amplification. Concurrently, new contrasted regions emerge along the structure, suggesting the activation of additional growth sites.

To elucidate the relationship between the contrasted islands and folding, we closely examine the formation of a representative fold initiated between two adjacent islands (Fig. 4e and Movie S3). At the initial stage, the whisker segment exhibits a relatively uniform thickness and a continuous SEI-shell with homogeneous contrast. Subsequently, two closely spaced contrasted regions emerge, characterized by locally thickened Li and a relatively thinner SEI-shell. These regions then begin to separate, defining a confined zone between them where deformation is initiated.

As illustrated schematically, Li deposition occurring from both directions at these sites generates compressive stress within the intervening region. This stress manifests as localized thickening and densification, consistent with the increased contrast observed experimentally. Between the two islands, a comparatively uniform growth region develops, and the increasing separation between the islands amplifies the accumulated compressive stress. Once a critical threshold is reached, this stress is relieved through the formation of a fold. An alternative stress-relief pathway is also observed in the form of twisting, indicating multiple modes of mechanical accommodation. Importantly, the thinning of the SEI shell under compressive stress creates favorable conditions for Li transport and insertion. This enables localized growth within the confined region, where opposing growth directions promote the development of a folded structure.

Overall, these observations demonstrate that contrasted islands act as stress localization centers that govern the initiation of folding through a coupled mechanism involving SEI thinning, localized lithium accumulation, and compressive stress–driven deformation.

To confirm the transition from diffusion-limited root growth to a distinct growth regime, a quantitative kinetic analysis was performed. Representative structures evolving concurrently were selected, including a loop and needle N-I, as well as a folding branch and a co-growing needle N-II (Fig. S3), and their growth kinetics (length *vs.* time) were compared (Fig. 4f). The results reveal a clear divergence in growth behavior. In the loop *vs.* N-I comparison (Fig. 4f, top), the needle length follows a square-root-of-time dependence 
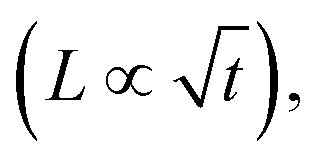
 consistent with classical diffusion-limited growth governed by Li supply through the SEI and root-based deposition, with an average growth rate of approximately 15 nm s^−1^. In contrast, the loop exhibits a markedly faster growth rate of approximately 207 nm s^−1^, growing approximately 14 times faster than N-I, and displaying a distinct kinetic signature indicative of a departure from diffusion-controlled behavior. Similarly, in the branch *vs.* N-II comparison (Fig. 4f, bottom), the folding branch grows at approximately 381 nm s^−1^, compared with approximately 34 nm s^−1^ for the co-growing N-II, *i.e.*, approximately 11 times faster, while N-II maintains a diffusion-limited trend.

This pronounced kinetic acceleration provides quantitative evidence that the onset of deformation and folding is accompanied by a transition from conventional diffusion-limited root growth toward a distinct local growth regime. This transition strongly correlates with the emergence of contrasted islands and the formation of cracks within the SEI shell, which create new electrochemically active sites for local Li deposition. Together, these observations support a stress-assisted, multi-site growth mechanism in which SEI disruption and localized Li transport accelerate dendrite growth and drive folding and structural reconfiguration.

**Fig. 5:**
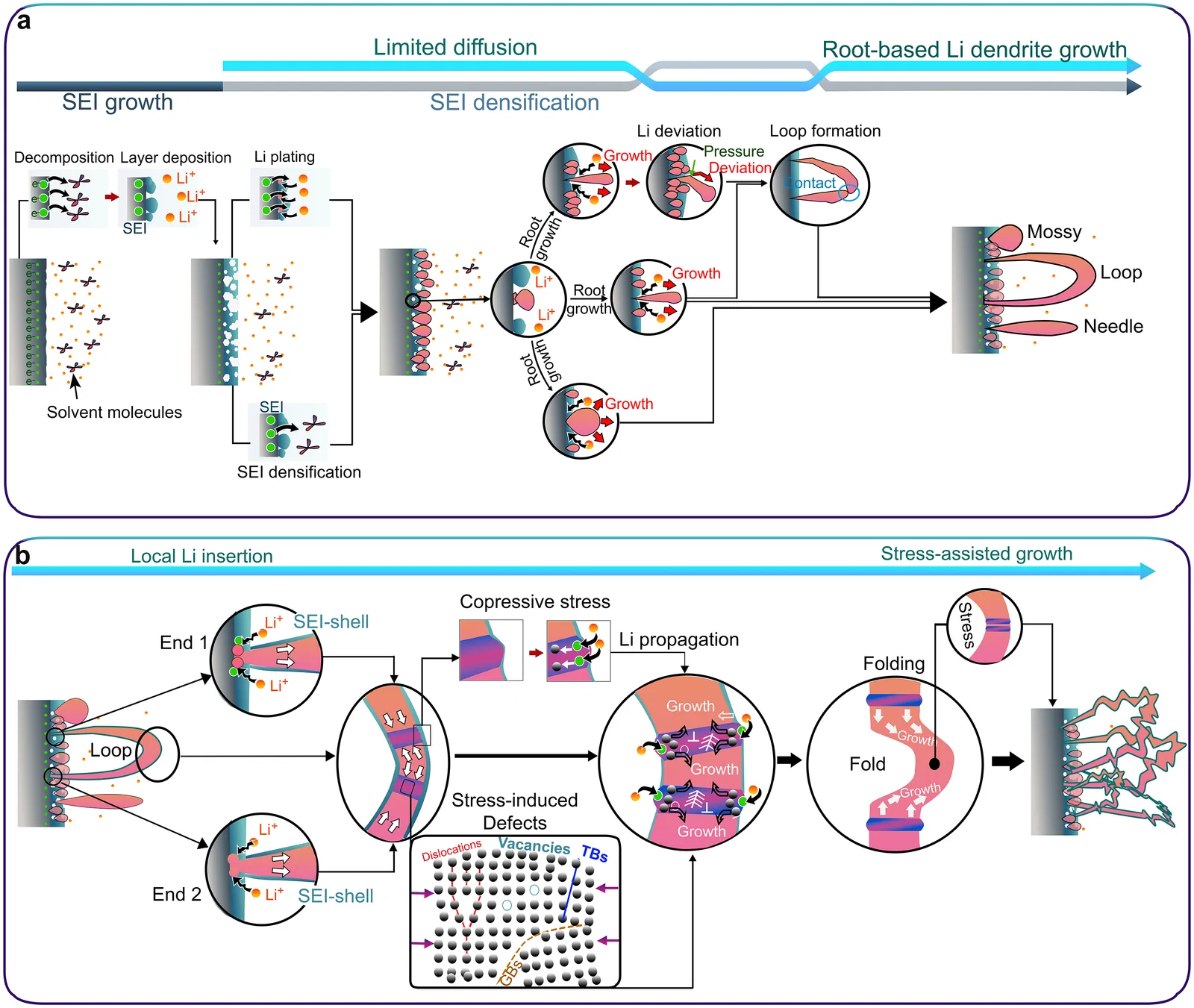
Schematic illustration of the transition from diffusion-limited root growth to stress-assisted, multi-site growth of lithium dendrites, as revealed by *in situ* ec-LC-TEM. (a) Diffusion-limited root growth involving SEI formation/densification, whisker deviation, and loop formation. (b) Stress-assisted growth driven by localized Li insertion, defect accumulation, SEI cracking, folding, and multidimensional dendritic evolution.

The present *operando* ec-LC-STEM observations enable real-time tracking of the growth and evolution of Li dendrites, providing direct insight into the mechanisms governing their deformation. Our results demonstrate that dendrite formation cannot be adequately described by conventional root- or tip-growth models alone. Instead, our observations reveal that, beyond the initial electrochemical and transport-controlled growth, subsequent dendrite evolution is strongly influenced by mechanical interactions between neighboring structures in conjunction with SEI dynamics. In particular, the intrinsic plasticity of Li plays a central role, enabling stress accommodation through deformation and thereby controlling the morphological evolution of dendrites within the cell. Fig. 5a and b schematically summarize the growth and evolution mechanisms of Li dendrites across length scales, from the nanoscale to the atomic scale. Based on the present study, the overall mechanism can be divided into two principal stages: (i) diffusion-limited root-based growth governed primarily by SEI-controlled Li^+^ transport (Fig. 5a), and (ii) stress-assisted growth governed by local Li deposition, mechanical interactions, and structural reconfiguration (Fig. 5b).

During the charging process, the low electrochemical potential of freshly deposited lithium metal induces the reduction of the electrolyte, leading to the formation of an initially incomplete and heterogeneous SEI layer at the electrode surface. This non-uniform SEI permits continued contact between the electrolyte and the electron-rich GC edge, allowing local Li deposition.^25^

Li initially nucleates as small spherical deposits. Subsequent growth proceeds *via* a root-based mechanism, in which continued Li deposition at the base pushes these spheres outward toward the electrolyte. This process gives rise to a variety of Li morphologies, including needle-like structures, mossy deposits, and whiskers, consistent with many previous reports.^35^ As the SEI progressively densifies, it increasingly limits Li ion transport to the root of the growing structures. Regions where the SEI remains incomplete or more permeable allow enhanced Li ion access, leading to preferential growth and the formation of elongated needles. The morphology and kinetics of these electrodeposited Li structures are intrinsically coupled to the local electrochemical conditions. Current density and electrode overpotential influence Li-ion transport, nucleation, and subsequent growth, while strong transport limitations can generate concentration gradients that promote morphological instability, as commonly described within the framework of Sand-type behavior.^36^ In the present experiments, however, Li deposition is driven by cyclic voltammetry rather than under constant-current conditions; therefore, the instantaneous current and local current density evolve continuously with the applied potential and changing electrode morphology. Accordingly, the initial growth behavior reflects the combined influence of electrochemical driving forces and mass transport. As the elongated Li structures develop, however, SEI confinement and interactions with neighboring deposits introduce additional mechanical constraints that progressively influence their subsequent evolution.

As these mechanical constraints develop, stress accumulation induces directional deviations of the needles, leading to the formation of necked regions. As growth proceeds, neighboring Li structures can interact through various contact configurations, including tip–tip, tip–side, and side–side interactions. Owing to the high surface-to-volume ratio of these nanoscale Li structures and the high homologous temperature of lithium, such contacts can be stabilized through surface diffusion–driven coalescence.^37^ This process minimizes overall surface energy and promotes diffusion bonding between adjacent structures. Consequently, the combined effects of SEI heterogeneity, mechanical confinement, and surface-energy minimization drive the transition from isolated needle growth to interconnected, mechanically coupled loop-like networks. These observations are consistent with the mechanism proposed by Becherer *et al.*, who suggested that parallel Li needles can meet and connect.^38^ Importantly, our high spatio-temporal resolution imaging provides direct experimental evidence supporting this mechanism while further revealing that these interactions are preceded and enabled by stress-induced structural deviations prior to physical contact. At this stage, growth remains predominantly diffusion-limited, as the SEI-shell surrounding the Li structures restricts Li ion transport to the reaction sites. As a result, Li deposition occurs mainly at the root, where the electrolyte–SEI interface enables electron transfer, leading to relatively slow kinetics governed by mass transport through the SEI. However, once loop structures are formed, their dual active growth fronts enable Li deposition from opposing ends, thereby accelerating overall growth kinetics relative to isolated needle structures (Fig. 5b).

Bidirectional growth from the two ends generates opposing forces along the loop segments, producing coupled compressive and tensile stresses (Fig. 5b). These stresses are localized at regions of curvature and kinks, where stress concentration drives plastic deformation. At these sites, defect density increases primarily through the generation and accumulation of dislocations, which can reorganize into sub-grain (low-angle) boundaries and, under continued strain, evolve toward higher-angle boundaries; local lattice rotations and deformation bands may also develop.^39^ Confinement by the SEI likely limits the escape of these defects from the Li core, promoting their accumulation in highly localized regions and thereby creating preferential pathways for Li atom transport.^40^ Concurrently, localized mass accumulation leads to segment thickening, which further amplifies the local stress field and imposes increasing mechanical loading on the surrounding SEI shell, ultimately promoting SEI thinning, rupture, and cracking. This disruption creates direct contact between the Li metal (electron source) and the electrolyte (Li^+^ source), generating newly exposed electrochemically active regions where localized Li^+^ reduction can occur beyond the original root. In parallel, the high defect density facilitates internal Li atom transport (*e.g.*, along dislocation cores and boundaries), enabling efficient mass redistribution within the structure. Together, these coupled processes establish a stress-assisted, multi-site growth mechanism that drives rapid deformation, folding, and structural reconfiguration of Li dendrites.

At stressed regions (kink), Li atoms redistribute along opposing directions, enabling growth from both sides of the segment. When similar stress localization occurs at neighboring kinks, this bidirectional growth generates opposing forces that promote folding as a plastic deformation response. The resulting folds further concentrate stress at specific locations, triggering additional defect accumulation and SEI cracking, which in turn activate new growth sites. This cyclic process leads to successive folding events and hierarchical structural evolution. At higher levels of deformation, a saturation regime is reached in which further stress accommodation occurs through twisting and complex three-dimensional reconfiguration. The absence of fracture and the dominance of folding and twisting indicate that Li accommodates stress primarily through plastic deformation. This behavior is consistent with the low yield strength of Li metal and is further enhanced by mechanical confinement imposed by the surrounding SEI.^41^

*In situ* observations provide further insight into the local growth behavior of loops in contact with neighboring needles. Once a loop is formed, newly nucleated needles emerge and grow until they interact with the loop at random locations. These interactions act as localized tensile forces, effectively generating a nano-indentation that pushes the loop from the point of contact while simultaneously inducing a pulling force on the opposite side. At the contact point, the needle and loop become mechanically and structurally connected. The resulting tensile stress can lead to partial detachment of the loop from one end, while the newly formed junction serves as a branching point, giving rise to multidimensional loop structures. This process occurs repeatedly due to the dynamic growth of both needles and loops, ultimately leading to the formation of a complex three-dimensional Li network. Such interconnected architectures, characterized by multiple active ends, further amplify mechanical interactions and promote additional deformation pathways during continued growth. Consequently, the system undergoes a transition from diffusion-limited root growth to a stress-assisted, multi-site growth regime, resulting in accelerated Li dendrite growth. This transition increases active lithium consumption and promotes the formation of electrochemically inactive (“dead”) Li during subsequent cycling.

Interestingly, in one of our previous studies,^25^ we demonstrated that SEI-shell thinning and cracking can directly trigger branch formation by creating localized electrochemically active sites on the Li surface, where Li ions gain access to electrons and are reduced to Li atoms that deposit outward, leading to surface protrusion and dendritic branching. In that mechanism, SEI failure primarily acted as a gateway for localized surface plating, enabling the transition from root growth to tip- or branch-driven structural evolution. In the present study, however, we reveal a fundamentally different consequence of SEI disruption under mechanically stressed conditions. Here, SEI thinning and cracking occur predominantly at highly deformed regions such as kinks and folds, where the underlying Li metal already contains a high density of plasticity-induced defects, including dislocations, sub-grain boundaries, and local lattice distortions. Under these conditions, the newly generated Li atoms formed at SEI-cracked regions are not preferentially deposited outward to form branches. Instead, local stress gradients and defect-rich pathways promote internal Li atom diffusion and mass redistribution within the existing Li structure, leading to localized thickening, folding, and structural reconfiguration. A critical distinction therefore emerges: while SEI cracking in relatively low-defect regions promotes outward surface growth and branch formation, SEI cracking in highly stressed, defect-rich regions instead drives defect-mediated internal growth that accommodates stress through plastic deformation. This distinction demonstrates that SEI rupture alone does not dictate dendrite morphology; rather, the local electrochemical–mechanical state of the Li structure governs whether Li evolves through branching or through stress-assisted folding and multidimensional structural evolution.

The mechanical evolution of Li dendrites has also been addressed in previous *operando* imaging studies. In 2014, Steiger *et al.* used *in situ* light microscopy to identify two characteristic modes of Li filament growth: growth from the base and localized growth between successive kinks. Their observations suggested that growth at kinked regions could be associated with crystallographic defects, while complementary SEM analysis revealed a clear connection between kinked Li needles and the formation of loop-like structures. However, the spatial resolution of optical microscopy did not allow the local processes underlying these mechanically induced morphological changes, particularly the formation and evolution of loop-like structures, to be directly resolved.^42^ Becherer *et al.* subsequently extended this picture by showing that Li can evolve into structures containing multiple kinks and remain connected to the electrode at both ends, thereby forming loop-like configurations. Based on previous literature, they proposed that Li transport and insertion at grain boundaries associated with kinked regions could contribute to the continued evolution of these structures.^38^ Nevertheless, the local mechanism connecting mechanical deformation, kink formation, SEI disruption, Li insertion, and the subsequent folding of the loops remained unresolved. The present work bridges this mechanistic gap by directly resolving the sequence linking these processes. Our *operando* observations show that initially root-growing Li whiskers undergo mechanically induced deviations and interactions, leading to contact, coalescence, and loop formation. Once mechanically constrained, continued growth generates localized deformation at kinks and folds, accompanied by SEI thinning and cracking. These mechanically generated SEI cracks create new electrochemically active regions for local Li penetration, while the defect-rich Li structure facilitates mass redistribution. Consequently, mechanical interaction is not merely a morphological consequence of dendrite growth but becomes directly coupled to subsequent Li deposition, redirecting the growth pathway and promoting folding and multi-site structural evolution. Importantly, this transition is also accompanied by a pronounced change in growth kinetics, providing quantitative evidence that the mechanically constrained structures evolve through a distinct growth regime. Thus, rather than considering root growth, kink formation, loop formation, SEI fracture, and plastic deformation as separate phenomena, our results connect them within a unified local electrochemo-mechanical mechanism describing the transition from diffusion-limited growth toward stress-assisted Li deposition.

Although the liquid-cell geometry does not reproduce the complete mechanical environment of a practical battery, the observed behaviour provides insight into the local response of Li deposits to mechanical confinement. In a conventional cell, growing Li structures interact with neighbouring deposits, the SEI, and the separator while being subjected to stack pressure. These constraints are expected to modify the magnitude and distribution of local stresses and therefore the resulting morphology and growth kinetics. Nevertheless, the present observations demonstrate a more general mechanism whereby obstruction of an actively growing Li structure generates local mechanical stress, promotes bending or buckling, and can redirect subsequent Li insertion toward newly activated sites. Separator confinement and stack pressure may therefore modulate, rather than eliminate, this stress–growth coupling. Overall, these observations reveal that Li dendrite evolution within batteries is a complex electrochemical–mechanical process strongly influenced by electrolyte chemistry, SEI dynamics, materials selection, and operating conditions. Beyond simple deposition, the intrinsic plasticity and stress-driven deformation of Li play central roles in shaping dendrite morphology, active Li consumption, and dead Li formation. Such mechanistic insight is essential for designing battery systems that suppress complex dendritic architectures, improve long-term performance, and reduce safety risks, particularly as Li plasticity may facilitate dendrite penetration through separators and promote internal short circuits.

## 4. Conclusion

Using *operando* ec-LC-STEM, we resolve the nanoscale mechanisms of Li deposition at the anode–electrolyte interface. We show that dendrite growth cannot be described solely by root- or tip-driven models, but instead evolves through a coupled electrochemical–mechanical pathway involving SEI densification, Li–Li interactions, and stress-induced deformation. Initial root-based growth produces needle-like structures that interact to form loops with dual active ends. Bidirectional Li insertion generates compressive stress, which is accommodated through plastic deformation (kinking, buckling, and folding) rather than fracture. Concurrently, SEI thinning and cracking create new insertion sites, while defect formation within Li enables internal mass transport, driving a transition from diffusion-limited to stress-assisted, multi-site growth. This mechanism leads to complex folded morphologies, accelerating Li consumption and the formation of inactive (“dead”) Li, which will be the subject of ongoing study. These findings provide a unified framework linking electrochemistry, mechanics, and interfacial processes, and highlight pathways to mitigate dendrite formation through targeted SEI and electrolyte design.

## Author contributions

W. D. conceived the idea and wrote the manuscript. W. D. fabricated the liquid cells, performed the liquid-cell experiments and realized the *in situ* STEM characterizations. W. D. carried out the data analysis. R. E., C. B. and R-S. K. contributed to the manuscript. All authors discussed the results and commented on the manuscript.

## Conflicts of interest

There are no conflicts to declare.

## Supplementary Material

MH-013-D6MH01280A-s001

MH-013-D6MH01280A-s002

MH-013-D6MH01280A-s003

MH-013-D6MH01280A-s004

## Data Availability

The data supporting the findings of this study are available from the corresponding author upon reasonable request. All data necessary to evaluate the conclusions presented in this paper are included within the article and its supplementary information (SI). Supplementary information: details for the experimental procedures, and Fig. S1–S3 (PDF). Captions for movies: Time-resolved Li metal deposition showing dendrite growth, loop formation, and subsequent folding evolution. (AVI). Time-resolved ADF-STEM imaging of Li dendrite folding and deformation dynamics. (AVI). Time-resolved ADF-STEM imaging of a Li branch showing the emergence of contrasted islands associated with folding initiation (AVI). See DOI: https://doi.org/10.1039/d6mh01280a.
