# Supplementary material for: Local mechanical behavior and stress-driven evolution of lithium dendrites revealed by operando electrochemical liquid-cell TEM
Source: Mater Horiz. 2026 Sep 2;13(20):10293–304. doi: 10.1039/d6mh01280a (PMC13536983; doi:10.1039/d6mh01280a)
Supplement: Supplementary file 1 [file MH-013-D6MH01280A-s004.pdf]

Supplementary Information (SI) for Materials Horizons.
This journal is © The Royal Society of Chemistry 2026

Local Mechanical Behavior and Stress-Driven Evolution of Lithium Dendrites Revealed by Operando Electrochemical Liquid-Cell TEM

Walid Dachraoui[a*], Ruben-Simon Kühnel[b], Corsin Battaglia[bcde], Rolf Erni[ae*]

[a]Electron Microscopy Center, Empa—Swiss Federal Laboratories for Materials Science and Technology, Überlandstrasse 129, 8600 Dübendorf, Switzerland.

[b]Materials for Energy Conversion, Empa—Swiss Federal Laboratories for Materials Science and Technology, Überlandstrasse 129, 8600 Dübendorf, Switzerland.

[c]Department of Information Technology and Electrical Engineering—ETH Zürich, Gloriastrasse 35, 8092 Zürich, Switzerland.

[d]Institute of Materials, School of Engineering – EPFL, Station 15, 1015 Lausanne, Switzerland

[e]Department of Materials – ETH Zürich, Wolfgang-Pauli-Strasse 10, 8049 Zürich, Switzerland.

Table of contents

Experimental Procedures

Supplementary figures: Figures S1 to S3

Captions for movies: Movie S1 to S3

Experimental procedures

1. Chemicals and consumables

A commercial LP30 electrolyte (1 M $LiPF_6$ dissolved in ethylene carbonate/ethyl methyl carbonate, EC/EMC = 3:7 v/v, battery grade, argon protected) was employed as the electrolyte medium throughout this study. Ferrocene (bis(cyclopentadienyl)iron, $Fe(C_5H_5)_2$) was obtained from Solvionic and used as received. Acetone, methanol, and anhydrous dimethyl carbonate (DMC, 99%) were purchased from Sigma-Aldrich without additional purification. Electrochemical liquid-cell microchips (E-chips) compatible with liquid-phase electron microscopy were supplied by Protochips. All reagents and consumables were handled according to manufacturer specifications unless otherwise stated.

2. Electrochemical liquid-cell transmission electron microscopy platform

2.1. Liquid-cell architecture and operando configuration

Operando electrochemical imaging was conducted using a closed-cell liquid-phase electron microscopy platform based on microfabricated silicon chips integrating electron-transparent silicon nitride (SiN_X) membranes. These membranes provide the mechanical resilience and chemical compatibility necessary for sustaining liquid electrolytes under high-vacuum electron microscopy conditions while preserving electron transparency for nanoscale imaging.

The experimental cell consisted of two complementary microchips assembled face-to-face to generate a sealed liquid compartment. The lower chip contained lithographically patterned SU-8 spacer structures positioned at the chip corners, defining the electrolyte thickness inside the chamber (50 nm nominal spacer height in this work). The upper chip incorporated the electrochemical circuitry, including Pt counter and reference electrodes, together with a glassy carbon (GC) working electrode positioned directly over the SiN_X imaging window to enable direct visualization of the electrode/electrolyte interface during electrochemical operation.

This three-electrode architecture was integrated into a Protochips Poseidon 510 holder equipped with microfluidic flow capability. Electrolyte transport into and out of the reaction chamber was achieved using syringe-pump-driven PEEK tubing, enabling controlled electrolyte exchange and minimizing contamination. Electrical connection between the chip electrodes and an external potentiostat was established through dedicated holder wiring routed along the TEM holder shaft. This design enabled synchronized electrochemical control and real-time imaging of interfacial processes, including SEI formation, lithium nucleation, and dendritic evolution.

2.2. Liquid-cell preparation and assembly procedure

Assembly of the electrochemical liquid cell was conducted through a sequential protocol designed to ensure reproducible cleaning, precise microchip alignment, and reliable sealing (Figure S1). Prior to assembly, both the electrochemical and spacer microchips were sequentially cleaned in isopropanol and acetone, followed by plasma cleaning to remove residual contaminants and improve surface wettability.[1]

Step 1: An elastomeric O-ring gasket was positioned within the designated holder cavity and inspected under optical microscopy to confirm proper placement and the absence of dust or particulates.

Step 2: The upper electrochemical microchip and lower spacer microchip were carefully aligned face-to-face to ensure accurate overlap of the viewing windows, electrode regions, and spacer structures, thereby defining the sealed electrochemical chamber.

Step 3: A metallic compression cover was then mounted and secured using screws to stabilize the assembled liquid cell, ensuring mechanical integrity and leak-tight sealing under electrolyte flow and TEM vacuum conditions.

This assembly process generated a hermetically sealed operando electrochemical platform capable of sustaining controlled electrolyte injection while preserving nanoscale imaging access through the SiN_X viewing window.

2.3. Device geometry and functional components

The removable silicon-based E-chips used in this work contained a 50 × 200 μm² electron-transparent SiN_X membrane with a nominal thickness of 50 nm. The lower chip integrated SU-8 spacer elements for liquid thickness control, while the upper chip hosted the lithographically defined electrochemical electrode network. The GC working electrode was positioned on the viewing membrane to maximize spatial correlation between electrochemical activity and electron beam interrogation.

This architecture enabled simultaneous structural, morphological, and interfacial characterization of electrochemical phenomena by allowing the electron beam to penetrate through the SiN_X membrane and liquid layer directly at the active working electrode.

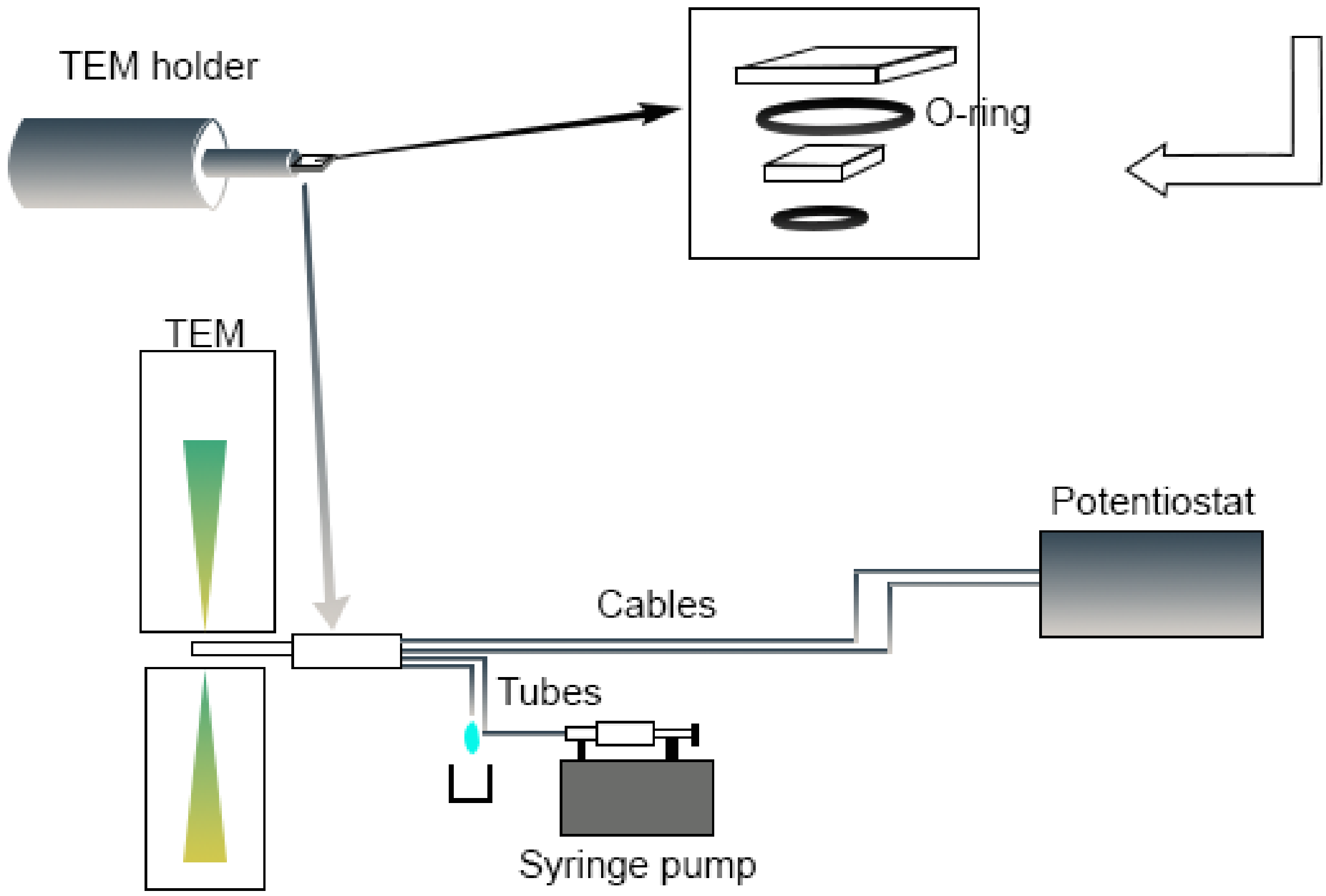

Fig. S1 Schematic illustration of the in situ ec-LC-TEM workflow. First, the electrochemical microchip and spacer microchip were sequentially cleaned in isopropanol and acetone for 2 min each, followed by 20 s plasma cleaning to remove surface contaminants and improve wettability. The two microchips were then carefully aligned face-to-face and assembled at the tip of the liquid-cell TEM holder using two O-ring gaskets to ensure proper sealing and liquid confinement. After holder assembly, the platform was inserted into the TEM column, where electrolyte was introduced into the sealed electrochemical cell using an external syringe pump connected

through microfluidic tubing. Electrochemical cycling was subsequently performed via an external potentiostat while operando imaging monitored dynamic processes at the electrode–electrolyte interface in real time.

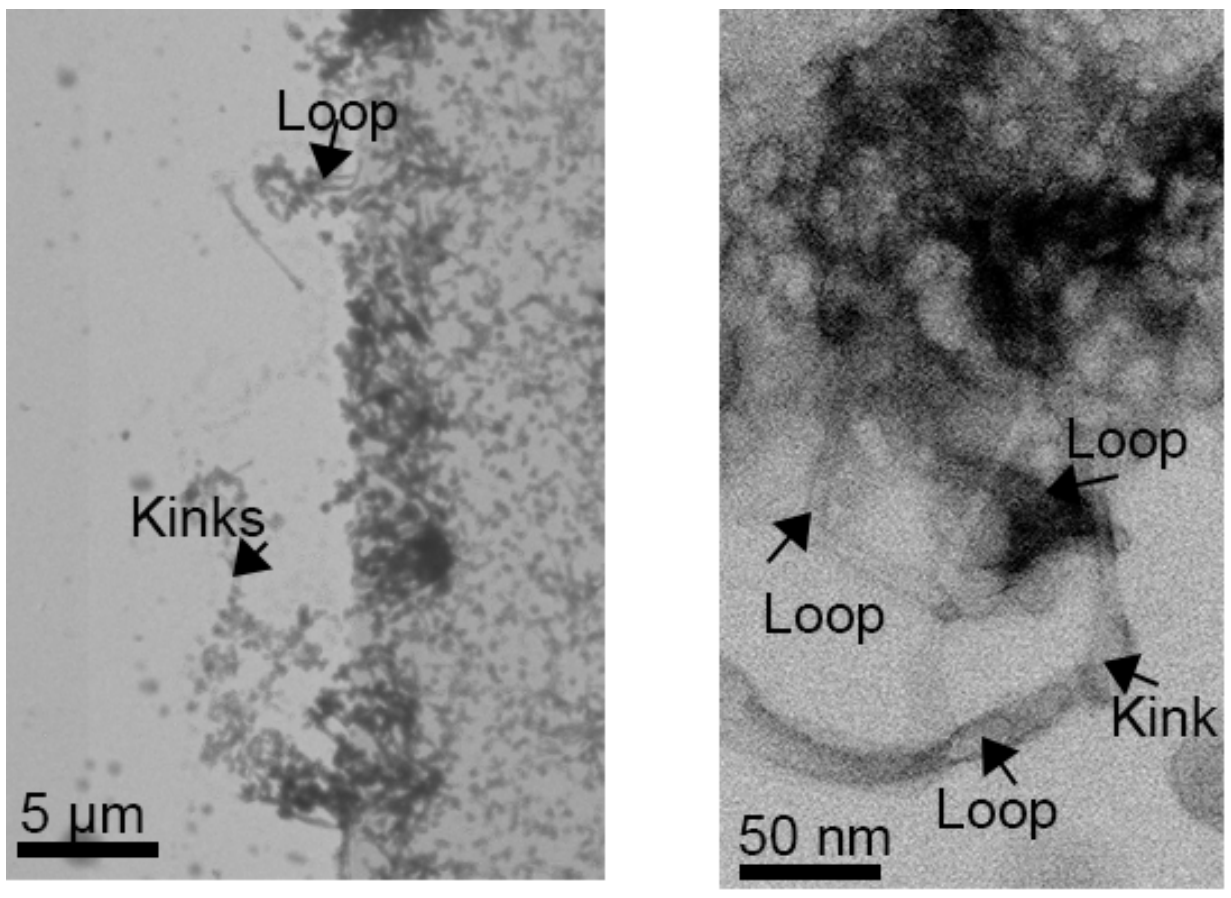

Fig. S2 ADF STEM images showin the existance of many loops and kinks witin the formed Li dendrites.

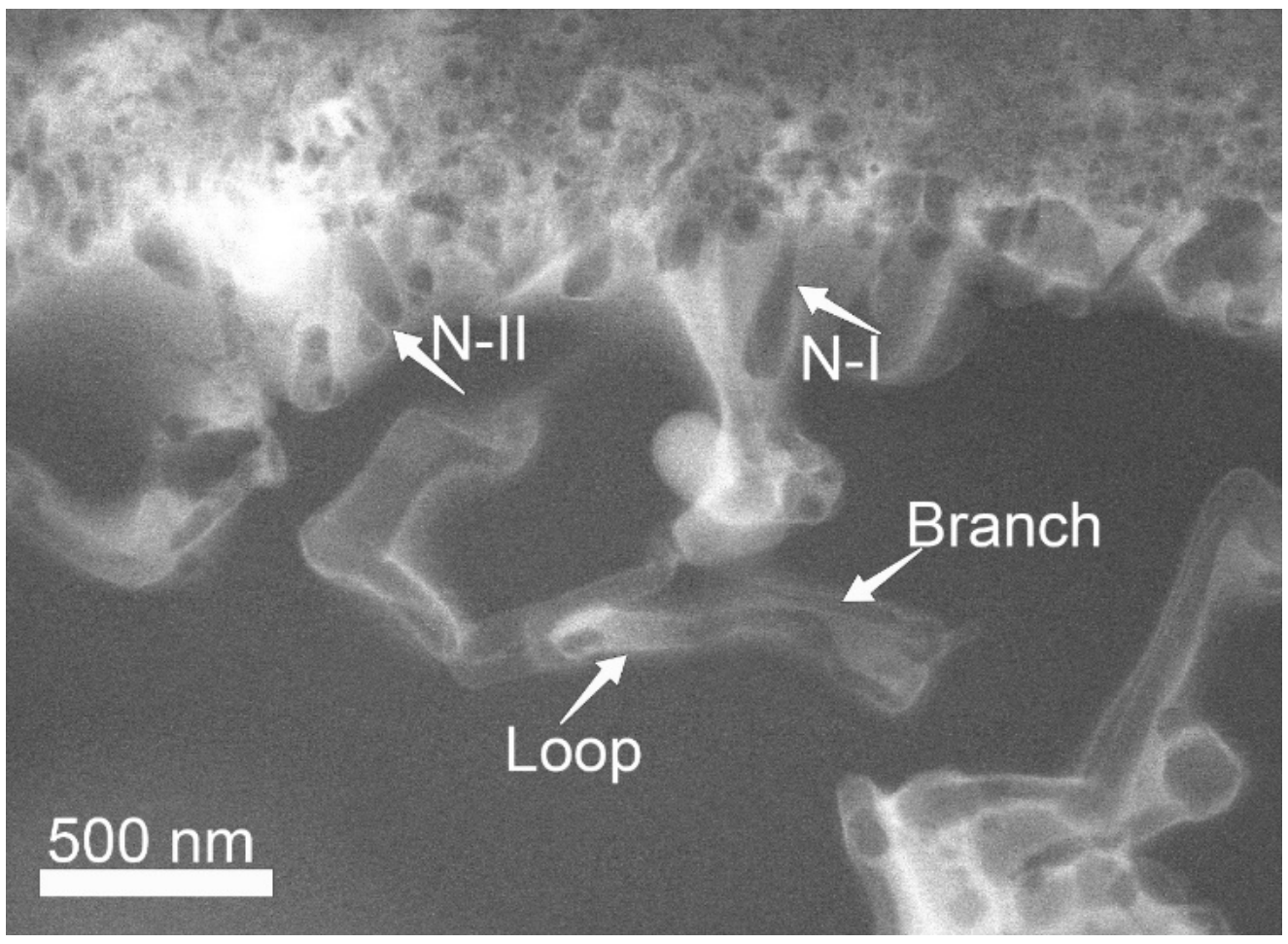

Fig. S3 ADF-STEM images showing the selected needles, loop, and branch structures used for the quantitative growth kinetics analysis presented in Figure 4f.

References

1 Dachraoui, W.; Pauer, R.; Battaglia, C.; Erni, R. Operando electrochemical liquid cell scanning transmission electron microscopy investigations of the growth and evolution of mosaic solid electrolyte interphase for lithium-ion batteries. ACS Nano 2023, 17, 20434-20444.

Captions for movies

Movie S1. Time-resolved Li metal deposition showing dendrite growth, loop formation, and subsequent folding evolution. Scale bar: 500 nm.

Movie S2. Time-resolved ADF-STEM imaging of Li dendrite folding and deformation dynamics. Scale bar: 200 nm.

Movie S3. Time-resolved ADF-STEM imaging of a Li branch showing the emergence of contrasted islands associated with folding initiation. Scale bar: 100 nm.
